# Enhancing Phytochemical Compounds, Functional Properties, and Volatile Flavor Profiles of Pomelo (*Citrus grandis* (L.) Osbeck) Juices from Different Cultivars through Fermentation with *Lacticaseibacillus paracasei*

**DOI:** 10.3390/foods12234278

**Published:** 2023-11-27

**Authors:** Vernabelle Balmori, Marisa Marnpae, Charoonsri Chusak, Kritmongkhon Kamonsuwan, Kasinee Katelakha, Suvimol Charoensiddhi, Sirichai Adisakwattana

**Affiliations:** 1 Center of Excellence in Phytochemical and Functional Food for Clinical Nutrition, Department of Nutrition and Dietetics, Faculty of Allied Health Sciences, Chulalongkorn University, Bangkok 10330, Thailand; balmorivernabelle@gmail.com (V.B.); mmarisa.hsc@gmail.com (M.M.); charoonsri.c@gmail.com (C.C.); kritmongkhon.kam@gmail.com (K.K.); 2 Department of Food Science and Technology, Southern Leyte State University, Sogod 6606, Southern Leyte, Philippines; 3 The Halal Science Center, Chulalongkorn University, Bangkok 10330, Thailand; kkasinee.hsc@gmail.com; 4 Department of Food Science and Technology, Faculty of Agro-Industry, Kasetsart University, Bangkok 10900, Thailand; suvimol.ch@ku.th

**Keywords:** pomelo juice, *Lacticaseibacillus paracasei*, lactic acid bacteria, flavonoids, volatile compounds

## Abstract

The current study aimed to explore the effects of fermenting five different pomelo cultivars using *Lacticaseibacillus paracasei* on various physicochemical, phytochemical, and organoleptic attributes. Fermentation led to an increase in viable lactic acid bacteria count (8.80–9.28 log cfu/mL), organic acids, total polyphenols, and flavonoids, resulting in improved antioxidant activity, bile acid binding, cholesterol micellization disruption, and inhibition of pancreatic lipase activity. Additionally, some cultivars displayed higher levels of naringin, naringenin, and hesperetin after fermentation. The levels of volatile compounds were elevated after fermentation. The bitterness and overall acceptability scores were improved in the fermented samples of the Kao Numpueng cultivar. The principal component analysis (PCA) revealed that the Tubtim Siam cultivar demonstrated the highest functionality and health-related benefits among all fermented pomelos. Overall, the study suggests that pomelo exhibits potential as a valuable resource for creating a dairy-free probiotic drink enriched with bioactive phytochemical compounds and beneficial functional attributes.

## 1. Introduction

Consuming foods that contain a variety of functional components has gained popularity due to their potential benefits for human health. Functional foods, such as those containing probiotics, antioxidants, fiber, prebiotics, and other secondary plant metabolites, are an emerging trend in the field of food science and nutrition [1]. Probiotics are one of the fastest-growing fields of functional foods because they have been found to exert potential health effects when consumed in sufficient quantities [2]. Remarkably, probiotics have exhibited a remarkable ability to modulate and influence the specific composition and balance of the gut microbiota. This modulation leads to various advantages such as decreased instances of gastrointestinal infections, enhanced lactose metabolism, immune system stimulation, potential protection against cancer, relief from diarrhea, and alleviation of symptoms associated with irritable bowel syndrome [3,4,5,6]. Probiotic foods have traditionally been dairy based, but there is increasing interest in exploring alternative non-dairy matrices as carriers of probiotic bacteria. This is due to concerns among certain populations, including those who are allergic to milk proteins, lactose intolerant, have issues with milk cholesterol, or are strict vegetarians [7]. Plant-based foods are excellent sources of vitamins, antioxidants, minerals, prebiotics, and bioactive compounds, which offer additional benefits to the product. Non-dairy probiotic foods include fruit juices fermented with probiotic strains, predominantly lactic acid bacteria. *Lactobacillus* and *Bifidobacterium* starter cultures have been primarily used to ferment different food matrices to develop functional products [8]. However, the use of these strains in fruit matrices is still being investigated due to technological challenges in maintaining the growth and survival of the probiotics, caused by intrinsic fruit characteristics, such as low pH and a lack of nitrogen sources [8]. *Lacticaseibacillus paracasei* has been identified as a suitable species for survival in juices like orange and mulberry with good shelf-stability at refrigeration temperatures [9,10]. Interestingly, fruit juices fermented by lactic acid bacteria can alter their composition due to the synthesis of new compounds or the release of bioactive components through microbial metabolism and biochemical reactions. This procedure enhances the physical, chemical, sensory, and functional attributes of the fruit structure [11]. Moreover, scientists are consistently investigating the impact of probiotic fermentation on enhancing the biological properties and health advantages of food items. This indicates that non-dairy probiotic foods have great potential for providing a wide range of health benefits to individuals with specific dietary needs and preferences.

Pomelo, scientifically known as *Citrus grandis* (L.) Osbeck, is a tropical fruit that originates from Southeast Asia. It is abundantly composed of vitamin C (ascorbic acid) and phytochemical compounds, such as polyphenols and flavonoids [12]. In Thailand, various pomelo varieties are locally grown, including Kao Yai, Tong Dee, Kao Tangkwa, Kao Numpueng, Ta Koi, and Tubtim Siam. Research has demonstrated that Thai pomelo contains varying amounts of bioactive compounds, such as flavonoids, which differ depending on the specific cultivar [13,14]. This variability makes it a promising raw material for producing non-dairy probiotic beverages. However, the type of pomelo cultivar used can influence the resulting phytochemical content and biological activity of the fermented beverage. Hence, this study aimed to produce a fermented drink by employing various Thai pomelo cultivars and *Lacticaseibacillus paracasei*. Moreover, the influence of fermentation on the physicochemical properties, probiotic viability, volatile compounds, biological and functional characteristics, as well as sensory acceptability of the beverage were also examined.

## 2. Materials and Methods

### 2.1. Raw Materials, Bacteria, and Chemicals

Five different cultivars of pomelo fruits, namely Kao Yai (KY), Kao Numpueng (KN), Tubtim Siam (TS), Tong Dee (TD), and Ta Koi (TK), were purchased from the Talaad Thai Fruit Market located in Pathum Thani Province, Thailand.

The freeze-dried powder of *L. paracasei* subsp. *paracasei* CASEI 431 (Chr. Hansen A/S, Horsholm, Denmark) was obtained from Brenntag Ingredients (Thailand) Public Company Limited, Bangkok.

De Man–Rogosa–Sharpe (MRS) agar was procured from Oxoid (Basingstoke, UK). Food-grade sodium bicarbonate was obtained from Thai Food and Chemical Co., Ltd. (Bangkok, Thailand). Folin–Ciocalteu’s reagent, phenol solution, sulfuric acid, potassium sodium tartrate, potassium dihydrogen phosphate, phosphoric acid, glucose standard, gallic acid, ascorbic acid, 1,1-diphenyl 2-picrylhydrazyl (DPPH), porcine bile extract, 6-hydroxyl-2,5,7,8-tetramethylchromane-2-carboxylic acid (Trolox), 2,4,6-tripyridyl-s-triazine (TPTZ), ferrous sulfate, hydrochloric acid, naringin, hesperidin, neohesperidin, naringenin, hesperitin, acetic acid, acetonitrile, oleic acid, bile extract, phosphatidylcholine, glycodeoxycholic acid (GDA), taurocholic acid, 4-methylumbelliferone, porcine pancreatic lipase, and sodium citrate were obtained from Sigma-Aldrich Co. (St. Louis, MO, USA). The cholesterol test kits (Cholesterol Liquicolor^®^) were purchased from Human Diagnostics (Wiesbaden, Germany), and the total bile acid assay kit was purchased from BIOBASE (Jinan, Shandong, China). An alkane mixed standard of C6–C16 used for volatile compounds analysis was obtained from Restek Corporation (Bellefonte, PA, USA).

### 2.2. Fermentation of Pomelo Juices

The pomelo juice was obtained by removing the peel, seeds, and skin from the fruits, followed by extracting the fruit pulp using a low-speed juice extractor (Buono, BUO-143536). This pulp was then mixed with drinking water at a ratio of 4:1 (*v*/*v*). To eliminate solid particles, the resulting juice was filtered using cheesecloth, and sugar (5%) and salt (0.05% *w*/*v*) were added. Pasteurization was conducted by subjecting the juice to a temperature of 80 °C for 30 s. The pasteurized juice samples were collected and stored at −20 °C as control (non-fermented) juices. For the fermented samples, the pH of the pomelo juice was adjusted to 6.0 using 1 M sodium bicarbonate. Subsequently, *L. paracasei* powder was directly introduced at an initial microbial concentration of 7 log cfu/mL, followed by incubation at 37 °C for 24 h [14]. Once the fermentation period concluded, the juices were rapidly cooled down and stored at −20 °C for subsequent testing. Each experiment was performed in triplicate.

### 2.3. Determination of pH and Color Properties

The pH of the juice samples was measured using a digital pH meter (Seven Compact S220, Mettler Toledo, Columbus, OH, USA). The color properties of the juice samples, including the values for L*, a*, and b*, were assessed utilizing a colorimeter (Colorflex, Hunter Associates Laboratory, Inc., Reston, VA, USA). The total color change (delta E) was calculated based on the changes in color values after fermentation. Each experiment was performed in triplicate.

### 2.4. Total Sugar and Reducing Sugar Content Analysis

The quantification of total sugar content in the samples was performed using the phenol-sulfuric acid method [15]. The fermented and non-fermented pomelo juices were diluted by adding distilled water at a 400-fold ratio. Subsequently, the diluted sample (400 µL) was combined with the working reagent in the glass tube (10 µL of an 80% phenol solution and 1 mL of a 95.5% sulfuric acid solution). Then, it was placed in a water bath set at 37 °C for 10 min. Following that, a period of 10 min was allotted for them to cool down to the ambient room temperature. The absorbance was then recorded at a wavelength of 490 nm. Each experiment was performed in triplicate.

The 3,5-dinitrosalicylic acid (DNS) method, as described by Jain et al. [16], was utilized to analyze the reduced sugar content. In this method, the juice sample (200 µL) was reacted with 200 µL of DNS reagent and subsequently boiled at 100 °C for 10 min. Once the mixture had reached a lower temperature, 200 µL of a 40% potassium sodium tartrate solution was introduced into the mixture. Subsequently, the measurement of absorbance was conducted at a wavelength of 540 nm. The glucose solutions were utilized to prepare a standard curve. Each experiment was performed in triplicate.

### 2.5. Analysis of Organic Acid

The quantification of organic acids was conducted using a well-established high-performance liquid chromatography (HPLC) method outlined in a prior publication [14]. First, the juice samples were subjected to centrifugation at 6500× *g* for 20 min at 4 °C. Subsequently, the supernatants were filtered through a 0.2 μm polyvinylidene fluoride syringe filter to prepare them for HPLC analysis. The mobile phase consisted of 10 mM potassium dihydrogen phosphate, pH 2.4, with phosphoric acid (A) and acetonitrile (B). The flow rate was set at 0.5 mL/min, and the gradient program consisted of the following: 0.01 min, 1% B; 12 min, 12% B; 15–24 min, 40% B; 25 min, 1% B, and held to 28 min, kept at 35 °C. Samples were injected at 5 µL per injection, and a mixed standard solution containing ascorbic, tartaric, lactic, citric, acetic, propionic, and malic acids was prepared to determine the retention times of each organic acid. External standards were used to quantify the peak area of the samples. Each experiment was performed in triplicate.

### 2.6. Enumeration of Viable Lactobacilli

To determine the total viable lactobacilli count, the standard plate count method was employed. The samples were plated on MRS agar and subjected to incubation at 37 °C for a duration of 48 h. Following incubation, the number of colony-forming units per mL was enumerated using the methodology described by a previous report [17]. Both analyses were performed in triplicate.

### 2.7. Determination of Phytochemical Compounds and Antioxidant Activity

The total phenolic content (TPC) was quantified by employing the Folin–Ciocalteau assay [18] and expressed as µg gallic acid equivalent (GAE) per mL of juice. The ferric-reducing antioxidant power (FRAP), DPPH radical scavenging activity, and Trolox equivalent antioxidant capacity (TEAC) were measured following the techniques described by Chayaratanasin et al. [19]. The FRAP values were presented as millimoles of ferrous sulfate (FeSO_4_) equivalent per mL of juice, while the DPPH radical scavenging activity was reported as milligrams of ascorbic acid equivalent (AAE) per mL. Additionally, the TEAC values were reported as milligrams of trolox equivalent per mL of juice. The quantification of the total flavonoid content (TFC) was conducted through a colorimetric assay, employing the methodology established by Adisakwattana et al. [20]. All analyses were carried out in triplicate and the results were expressed as micrograms of quercetin equivalent (QE) per mL of juice.

### 2.8. Quantification of Major Flavonoid Constituents

In this study, the impact of fermentation on flavonoid constituents was examined through the application of HPLC, following a previously established method with slight modifications [21]. To begin, freeze-dried juice samples were carefully weighed and combined with an aqueous methanol solution (50% *v*/*v*) prior to homogenization for 1 min. Subsequently, the mixtures were subjected to extraction by immersing them in a water bath set at a constant temperature of 30 °C for a duration of 20 min. Following the extraction process, centrifugation was performed at 4500 rpm at 20 °C for a duration of 20 min. The resulting supernatants were collected and subjected to solid-phase extraction (SPE) using a Sep-Pak C_18_ cartridge. The column was preconditioned with 6 mL of absolute methanol and 6 mL of 0.01 N HCl solutions, followed by the introduction of the supernatant into the cartridge and washing with 20 mL of 0.01 N HCl to remove sugars and other organic acids. Final washing was carried out with a methanol/HCl solution (1% *v*/*v*) to elute the retained compounds. Washings were collected and subsequently dried using a rotary evaporator at 50 °C and 150 rpm until the volume was reduced to approximately 0.5 mL. Further drying was performed under nitrogen, and completely dried extracts were reconstituted with a water/acetic acid (99:1, *v*/*v*) and acetonitrile/acetic acid (99:1, *v*/*v*) mixture (1:1) before being filtered prior to HPLC injection. The mobile phase was composed of (A) water/acetic acid (99:1, *v*/*v*) and (B) acetonitrile/acetic acid (99:1, *v*/*v*) at a column temperature of 50 °C. The gradient flow was set as follows: 0 min, 5% B; 5 min, 8% B; 7 min, 12% B; 12 min, 18% B; 17 min, 22% B; 22 min, 25% B; 27 min, 35% B; 37 min, 53% B; 38 min, 53% B; 40 min, 55% B; 42 min, 60% B; 57 min, 80% B; 60 min, 85% B; and 65 min, 85% B. The flow rate was set at 1.2 mL/min, and chromatographic detection was carried out at 280 nm. Identification and quantification were performed through the use of an external standard composed of naringin, naringenin, hesperidin, neohesperidin, and hesperetin. The analysis was performed in triplicate, and the results were expressed in µg/g of freeze-dried juice.

### 2.9. Bile Acid Binding, Cholesterol Micellization, and Pancreatic Lipase Inhibition

In the bile acid binding assay, the experimental procedure involved incubating the samples with bile extract (2 mM) and glycodeoxycholic acid (GDA) (2 mM) in 0.1 M phosphate-buffered saline (PBS) at a temperature of 37 °C for a duration of 90 min [13]. Subsequently, the mixtures underwent filtration and were subjected to analysis using a bile acid assay kit. Analysis was performed in triplicate and the results were then reported as the percentage of bile acid binding. For comparative purposes, a positive control was incorporated using cholestyramine (3 mg/mL).

In the cholesterol micellization assay, synthetic micelles were prepared by combining 2 mM cholesterol, 1 mM oleic acid, 2.4 mM phosphatidylcholine, and 6.6 mM taurocholic acid in 15 mM PBS [13]. The samples were introduced to the mixed micelles and subjected to incubation at 37 °C for a duration of 2 h. Finally, the mixtures were centrifuged at 10,000 rpm for 20 min. The cholesterol concentration in the supernatants was assessed using a cholesterol test kit. Gallic acid (0.5 mg/mL) was used for the positive control. The analysis was repeated 3 times and the results were expressed as the percentage of cholesterol micellization inhibition.

In the pancreatic lipase activity assay, the samples were mixed with a solution of 0.2 mM 4-methylumbelliferone (4-MUO) in a microplate well [13]. Subsequently, the pancreatic lipase enzyme solution (7.5 mg/mL) was added to the mixtures and incubated at a temperature of 37 °C for a duration of 20 min. Following that, 0.1 M sodium citrate solution at pH 4.2 was added to halt the enzymatic reaction. The measurement of lipase activity was conducted using a fluorescence microplate reader, with excitation occurring at 355 nm and emission detected at 460 nm. Analysis was conducted in triplicate and orlistat was employed as a positive control.

### 2.10. Analysis of Volatile Compounds by Gas Chromatography

The volatile flavor compounds in the juice samples were analyzed using an electronic nose operating on ultrafast gas chromatography with two columns (Hera-cles II, Alpha M.O.S., Toulouse, France), following a previously established method with slight modifications [14]. The instrument was equipped with two columns of different polarities, namely, nonpolar MXT-5 and slightly polar MXT-1701, and two flame ionization detectors (FID).

To begin the analysis, juice samples were carefully transferred into sealed vials and incubated at a temperature of 60 °C for 300 s. Throughout the incubation process, agitation was maintained at 500 rpm within the auto-sampler. The gas produced in the headspace was then analyzed by gas chromatography with an injection volume of 5 mL and collected in a trap at 25 °C before desorption in two columns at 250 °C. The oven temperature initiated at 40 °C for 10 s, then increased to 60 °C at 4 °C/s, and finally to 250 °C at 1 °C/s. The FID detectors were set at 270 °C. Alkane standards C6-C16 analyzed at the same condition served as reference standards to convert retention times into Kovats retention indices (KI) using AlphaSoft software v14.1 (Alpha Software Co., Burlington, MA, USA). Volatile compounds were tentatively identified by comparing their KI values with the AroChemBase v5.0 reference volatile compound database (Alpha MOS, Toulouse, France), considering the possibility of a match (relevant index) at more than 80% [14].

Through untargeted qualitative analysis, the peak areas of each identified compound were compared to evaluate the influence of fermentation on alterations in volatile compounds. The analysis was performed in triplicate, with three independent repetitions (injections) carried out for each individual sample.

### 2.11. Organoleptic Test

A group of 50 individuals without specific training evaluated the organoleptic acceptability of the samples using a 9-point hedonic evaluation test. The assessment encompassed different attributes, including color, aroma, sweetness, sourness, bitterness (aftertaste), and overall acceptability. The protocol for this test was approved by the Research Ethics Review Committee for Research Involving Human Research Participants, Chulalongkorn University on 18 April 2022 (Certificate of Approval No. 090/65, Study Title No. 650032). Before commencing the experiment, written informed consent was obtained from all panelists participating in the evaluation.

### 2.12. Statistical Analysis

The obtained data were presented as the mean ± standard error of the mean (SEM). To examine the effects of cultivar variation, fermentation, and their interaction, a two-way analysis of variance (ANOVA) was conducted. In cases where significant main effects were detected, further analysis was performed using Duncan’s Multiple Range Test (DMRT) to identify significant differences between groups. The paired t-test was utilized to determine the statistical significance of differences observed before and after fermentation for the parameters under investigation. The data were analyzed using IBM SPSS version 22.0 (Chicago, IL, USA), and the significance level was set at *p* < 0.05. The principal component analysis was conducted using the R software (Version 2023.09.1+494) to visualize the differences between the samples based on the identified factors.

## 3. Results and Discussion

### 3.1. Physicochemical Properties of Fermented Pomelo Juices

During this study, we made significant observations regarding the effects of fermentation on the pH levels and chemical composition of pomelo juices. After the fermentation process, the pH levels of the samples decreased notably, reaching a final pH range between 4.26 and 4.49, as indicated in Table 1. This decrease in pH can be attributed to the increased production of organic acids, particularly lactic acid and acetic acid. The rise in organic acids is directly responsible for the reduction in pH levels of fermented pomelo juices. Furthermore, this fermentation process led to a substantial decline in both the total and reducing sugar content of the juices obtained from all pomelo cultivars. The findings of our study revealed that the strain *L. paracasei* CASEI 431 played a key role in converting the glucose and fructose present in pomelo juice into lactic and acetic acid through an enzymatic process.

This enzymatic conversion resulted in a significant decrease in sugar content and a simultaneous increase in the organic acid content of the juice. Previous studies have also reported similar findings, demonstrating the ability of lactic acid bacteria to convert citric acid to lactate and acetate, resulting in reduced citric acid levels and higher lactic acid content [10,22]. The observed levels of organic acids in the non-fermented juices were lower than the typical organic acid content of pure pomelo juice as observed by a previous study [23] since the pomelo pulps were added with water in the juice extraction. Additionally, a previous study has observed that orange juice fermented with *L. paracasei* has led to an increase in lactic and acetic, as these acids are the major metabolites of LAB fermentation [9]. Pomelo fruits contain soluble sugars including glucose, fructose, and sucrose, which are utilizable by *L. paracasei* species, thus reducing the sugar content [24]. Fermentation of fruits and vegetables by lactic acid bacteria has been associated with enhanced health benefits due to the reduction in sugar content [25]. Similar outcomes have been observed in studies investigating the fermentation of other fruit juices, such as orange juice [9] and gac juice [14]. Interestingly, we found that the Tubtim Siam and Kao Yai cultivars exhibited the highest initial total sugar and reducing sugar content among all the cultivars studied (Table 2). This may explain their superior sweetness compared to other cultivars. Overall, our study underlines the transformative effects of fermentation on pomelo juice, leading to significant changes in pH, organic acid content, and sugar levels, which can impact the taste and potential health benefits of fermented juice.

The color of fruit juice plays a significant role in determining consumer acceptance, as it greatly influences sensory appeal and overall product quality [25]. In this study, the color values of fermented pomelo juice were evaluated, and it was found that the degree of lightness (L*) significantly increased for all cultivars after fermentation. Additionally, the a* values for Tong Dee, Ta Koi, and Tubtim Siam significantly increased after fermentation, indicating an increase in redness. Nevertheless, no significant difference in b* values was observed after fermentation for all cultivars. The alterations in juice color following fermentation may be attributed to the intricate relationship among pH, pigments, and color parameters. Pink varieties of pomelo, containing pigments like lycopene and anthocyanin, exhibit a more stable red color at pH < 4, consequently elevating the a* values of the juices. The reduction in lightness is linked to the oxidation and degradation of juice components during fermentation [26]. The calculated ΔE values across all cultivars (falling within the range of 4.31 to 5.26) suggest that the color changes post-fermentation are noticeable. It has been established that visual distinctions in color become apparent when ΔE exceeds 3 [27]. Consumers associate lighter-colored fruit juices with higher freshness and quality levels, which suggests that increasing lightness may improve consumer perception and acceptance of the product [26]. Therefore, it is possible that increasing the lightness of fermented pomelo by lactic acid bacteria may help to further improve consumer perception and acceptance of the product, although further research is needed to confirm this speculation.

### 3.2. Viable Lactobacilli Counts of Fermented Pomelo Juices

As demonstrated in Table 2, the viable lactobacilli count significantly increased after fermentation in all cultivars, with final counts ranging from 8.80 to 9.28 log cfu/mL, compared to an initial count of around 7 log cfu/mL. Among the cultivars, the highest viable counts were found in Tubtim Siam and Kao Yai, which are associated with a higher decrease in reducing sugar content in these cultivars. These findings indicate that pomelo cultivars are suitable carriers for *L. paracasei* bacteria, as they can proliferate in the juice matrix with viable counts higher than the recommended amount for beneficial effect (≥10^6^ cfu/mL) [2]. This study represents the initial exploration into the viability of lactobacilli bacteria in fermented pomelo juice. Our findings are in line with earlier studies that demonstrated the survival of lactobacilli bacteria in citrus juices, including grapefruit [28] and orange [9], highlighting the possibility of using citrus fruits as a nutrient-rich source for the proliferation of lactic acid bacteria.

### 3.3. Phytochemicals and Biological Activities of Fermented Pomelo Juices

The beneficial antioxidant activities of pomelo juice can be attributed to the presence of ascorbic acid and phytochemical compounds. The findings from our study demonstrated a significant increase in the TPC and TFC of all pomelo cultivars following fermentation with *L. paracasei* (Table 3).

Among the cultivars, Tubtim Siam exhibited the highest TPC and TFC levels both before and after fermentation, with values increasing from 1063 to 1288 μg GAE/mL and from 81.71 to 118.99 μg QE/mL, respectively. In addition, fermentation significantly increased the FRAP, TEAC, and DPPH scavenging activity of all the pomelo cultivars. The FRAP values of fermented samples were increased to 9.45–13.06 (mmol FeSO_4_/mL), which were 10.21–31.17% higher than the non-fermented juices. This suggests that fermentation with *L. paracasei* can improve the reducing power of pomelo juice. A similar outcome of increasing antioxidant activity was reported in a study conducted by Zhao and Shah [29] on fermented soymilk. Moreover, the TEAC and DPPH scavenging capacities of the pomelo juices exhibited a substantial increase subsequent to the fermentation process. The highest TEAC and DPPH capacities were observed in the fermented Tubtim Siam cultivar at 2.56 mg Trolox Eq/mL and 0.98 mg AAE/mL, respectively. Similar findings were reported in a study on fermented apple juice, indicating that lactic acid bacteria fermentation could enhance the accessibility of polyphenolic compounds characterized by their hydrogen-donating and oxygen-quenching properties [30]. We suggest that the variability in antioxidant activity among the different pomelo cultivars can be attributed to the varying levels of TPC and TFC, as well as the viability of *L. paracasei* in each cultivar.

Pomelo juice is abundant in phytochemical compounds, particularly belonging to the flavonoid family, specifically flavanones, which are the primary constituents. As presented in Table 4, the major flavanones presented in pomelo juice include naringin, which is the most abundant, followed by hesperidin and neohesperidin. A similar flavanone profile was reported in the study conducted by Makkumrai et al. [24]. Fermentation with lactic acid bacteria can transform conjugated flavonoid compounds into their free forms, which have improved bioavailability and higher antioxidant activity [31]. This results in enhanced health-related functionality of the flavonoids. Our findings indicated a significant increase in the levels of naringenin (52.83–91.37% increase) and hesperetin (39.16–341.18% increase) following fermentation. Naringenin is an aglycone of naringin and is naturally present in citrus fruits [28]. 

On the other hand, hesperetin is an aglycone of hesperidin and neohesperidin [32]. The increase in the content of aglycone flavonoids, which is associated with the rise in TPC and antioxidant activity, occurs as a result of the hydrolysis of glycosidic linkages by β-glucosidase enzymes produced by lactic acid bacteria [24]. This enzymatic process leads to the conversion of glycosylated flavonoids into their aglycone forms, which are more readily absorbed. By breaking down these glycosidic linkages, the lactic acid bacteria enhance the availability and bioactivity of flavonoids, thereby increasing the content of TPC and improving the antioxidant activity of the juice.

The findings show that the fermentation process of citrus fruits may lead to changes in the levels of certain compounds. Specifically, some cultivars experienced a decrease in the levels of hesperidin and neohesperidin after fermentation, but this decrease may not necessarily be negative, as the fermentation process could have led to the formation of other beneficial compounds. Interestingly, the levels of naringin, a flavonoid responsible for imparting bitterness to citrus fruits, increased in all cultivars after fermentation. The Tubtim Siam cultivar exhibited the highest naringin content among the tested cultivars (2271.38 and 2517.00 μg/g freeze-dried juice for non-fermented and fermented juice, respectively). The increase in naringin content observed in the fermented juices can be attributed to the degradation of citrus fruit cell walls that occurs during the fermentation process [33]. It is important to note that the complete conversion of naringin to naringenin may not occur during probiotic fermentation, likely due to the limited availability of β-glucosidase enzymes. Therefore, the increase in naringin levels following fermentation can be partially explained by the limited conversion of naringin to naringenin. The presence of naringin in the fermented juices contributes to the overall flavonoid content and may have potential implications for the sensory characteristics and health benefits of the final product. The naringin content in pomelo fruits can vary depending on the cultivar. Pink pomelo cultivars, such as Tubtim Siam, generally have higher levels of naringin compared to white varieties [34]. This difference in naringin content is likely due to genetic variations between the different pomelo cultivars. Therefore, when studying naringin levels in pomelo fruits, it is crucial to consider the specific cultivar being analyzed. Taking into account the specific cultivar of pomelo is important for accurately assessing and understanding the naringin content and its potential impact on the flavor and health benefits of the fermented juices.

### 3.4. Functional Properties of Fermented Pomelo Juices

**Figure 1:**
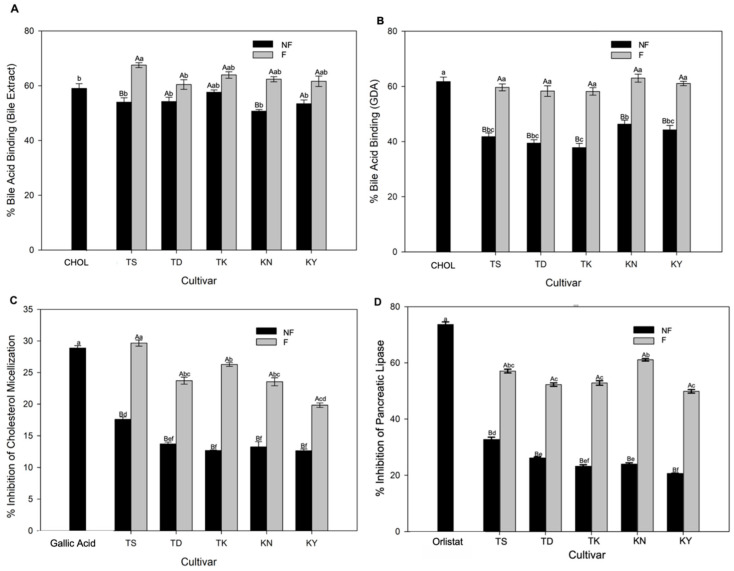
Effects of fermentation on bile acid binding (**A**,**B**), cholesterol micellization inhibition (**C**), and the pancreatic lipase inhibitory activity (**D**) of pomelo juices from different cultivars. Results are expressed as mean ± S.E.M. (*n* = 3). The means with different uppercase letters (fermentation effects) at the same cultivar are significantly different (*p* < 0.05). The means with different lowercase letters (treatment effects) are significantly different (*p* < 0.05). The abbreviations used for different cultivars are as follows: TS: Tubtim Siam; TD: Tong Dee; TK: Ta Koi; KN: Kao Numpueng; KY: Kao Yai; CHOL: Cholestyramine (3 mg/mL); Gallic acid at 0.5 mg/mL; Orlistat at 0.01 mg/mL; NF: Non-fermented juice; F: Fermented juice.

The results from Figure 1 show how fermentation affects the functional properties of pomelo juice, specifically its ability to bind bile acids, inhibit cholesterol micellization, and inhibit lipase activity. The study found that all varieties of pomelo juice could bind primary (bile extract) and secondary bile acids (GDA), showing binding capacities ranging from 50.71% to 57.62% and 37.80% to 46.33%, respectively (Figure 1A,B). This ability is due to the presence of polyphenolic substances in pomelo juice that can interact with bile acids through ionic, hydrogen, and hydrophobic interactions, forming polyphenol–bile acid complexes [35]. Fermentation significantly improved the capacity of pomelo juice to bind bile acid, with a range of 60.43% to 67.51% for primary bile acid and 58.30% to 62.98% for secondary bile acid (GDA). The binding values of fermented pomelo juices were found to be similar to those of cholestyramine.

In addition to its remarkable bile acid binding capacity, fermented pomelo juice exhibited an enhanced capability to disrupt cholesterol micellization formation. The increase in cholesterol micellization formation inhibition was significantly higher in the fermented juice compared to the unfermented juice (Figure 1C). Based on the findings, we suggest that this inhibitory action can be attributed to the bile acid binding activity of pomelo juices and the presence of lactic acid bacteria. Citrus juices, containing soluble fiber like pectin, can bind cholesterol, thereby exhibiting a hypocholesterolemic effect [36]. Meanwhile, *L. paracasei*, belonging to the species of lactic acid bacteria, is recognized for assimilating cholesterol and binding it to their cell walls, leading to reduced solubility and disruption of cholesterol micellization [37]. Furthermore, the fermented pomelo juice showed a higher inhibition of pancreatic lipase activity (Figure 1D), indicating that the presence of polyphenols might be responsible for this effect. These compounds have been shown to form a complex with lipase through hydrophobic associations and hydrogen bonding, causing a conformational change in the enzyme that inhibits its binding to the substrate, leading to its inhibition [38]. Among the cultivars, Tubtim Siam generally exhibited the highest functional properties related to disrupting lipid absorption, which could be attributed to its higher phytochemical content and LAB count compared to the other cultivars. The increased functional properties observed in this study may be attributed to the rise in total phenolic and flavonoid content of the fermented pomelo juice. The inhibitory mechanisms of fermented pomelo juices may have the potential to improve lipid metabolism related to fat absorption, thereby lowering the risk of conditions such as hypercholesterolemia and obesity [39]. However, further experiments are warranted to investigate the effects of fermented pomelo juice on bile acid binding capacity in humans.

### 3.5. Volatile Compounds of Fermented Pomelo Juices

The tentative identification of volatile flavor compounds greatly influences the quality of citrus fruits, contributing to their complex aroma. The identification was carried out in both fermented and non-fermented pomelo cultivars using KI compared to the database (Table S2). Forty volatile compounds were tentatively identified in fermented and non-fermented pomelo juice samples. Among these, thirteen compounds are presented in Table 5, including nine compounds (1-Hexanol, 1-octen-3-ol, (*Z*)-3-hexenal, 2,4-decadienal, (*E*,*E*)-, ethyl acetate, myrcene, (+)-alpha-phellandrene, L-limonene, and indole) that were previously identified in pomelo juices [23,40,41].

The major compounds are associated with fruity flavors (ethyl acetate), citrus flavors (including L-limonene, myrcene, and 2,4-decadienal (*E*,*E*)-), and ‘green’ flavors (bis(2-furylmethyl) sulfide). As reported by Rosales and Suwonsichon [34], pomelo fruits contain a variety of volatile compounds, including hydrocarbons, alcohols, esters, aldehydes, and minor components that contribute to a fruity, citrusy, and herbaceous aroma (green).

Fermentation of pomelo juice resulted in an increase in total ketones, including butan-2-one, acetoin, and 3,5-octadien-2-one, which were not found in non-fermented pomelo juices. Lactic acid bacteria, such as *L. paracasei*, can metabolize citric acid and pyruvate, producing acetoin and butanediol, with butan-2-one being produced through the dehydration of 2,3-butanediol [22]. During the fermentation process of pomelo juice, some undesirable compounds (green odors), such as 2,4-decadienal, (*E*,*E*)-, (*Z*)-3-Hexenal, and bis(2-furylmethyl) sulfide, were significantly reduced (*p* < 0.05). Unsaturated aliphatic aldehydes such as 2,4-decadienal are known to be aroma-active compounds present in both white and pink pomelo varieties, contributing to their citrus, green aroma. The reduction in the intensity of these compounds is attributed to the reduction or oxidation of aldehydes by lactic acid bacteria during fermentation, resulting in the formation of alcohols or carboxylic acids [42]. After the fermentation process, there was a notable decrease in the presence of undesirable aromatic compounds, including l-limonene. L-limonene, a terpene hydrocarbon naturally present in citrus fruits, contributes to the citrusy aroma of pomelo juice, while trimethylamine and 1-butanamine are associated with strong unpleasant fishy and ammonia-like odors [43]. High concentrations of terpenes and terpenoids in citrus fruits have been linked to high degrees of bitterness, which can affect consumer preference.

Based on our findings, it can be inferred that the fermentation process involving *L. paracasei* has the potential to enhance the volatile aromatic profile of pomelo juice. This improvement is attributed to the reduction of undesirable compounds and the enhancement or synthesis of desirable flavor compounds. Moreover, our results indicate that different cultivars of pomelo exhibit varying aromatic compound profiles, which may contribute to differences in aroma, flavor, and other organoleptic characteristics.

An electronic nose system, utilizing ultrafast GC, has been employed for food quality control and to investigate the influence of specific factors on changes in volatile compounds in food [44,45]. This method entails the simultaneous analysis of chromatographic profiles from two GC columns with different polarities, providing a comprehensive exploration of the volatile profile in various foodstuffs. Notably, it offers a quicker and simpler alternative to other chromatographic techniques.

The tentative identification, achieved through Kovats relative retention indexes on the two columns of different polarities, is designed to enhance accuracy. However, the study has limitations as it relies solely on tentative identification through KI from a library database, lacking additional confirmation using MS spectra. Therefore, further research is imperative, utilizing GC-MS/MS for more precise identification. In particular, volatile compounds not found in pomelo juice but emerging after fermentation, such as butan-2-one, acetoin, and 3,5-octadien-2-one, should undergo further identification via GC-MS to confirm and elucidate how fermentation influences the volatile profile of the juices.

Furthermore, GC-MS/MS facilitates the quantification of volatile compounds using internal standards, enabling the examination of concentrations in pomelo juice and the assessment of how fermentation affects them. Additionally, to investigate how odor compounds impact the aroma of pomelo juice, further analyses are required. This includes odor threshold testing and the evaluation of odor activity values (OAV), determining the minimum detectable concentration of odor compounds by human olfactory receptors, and assessing their overall impact on the aroma profiles of pomelo juice.

### 3.6. Organoleptic Acceptability Scores of Fermented Pomelo Juices

**Figure 2:**
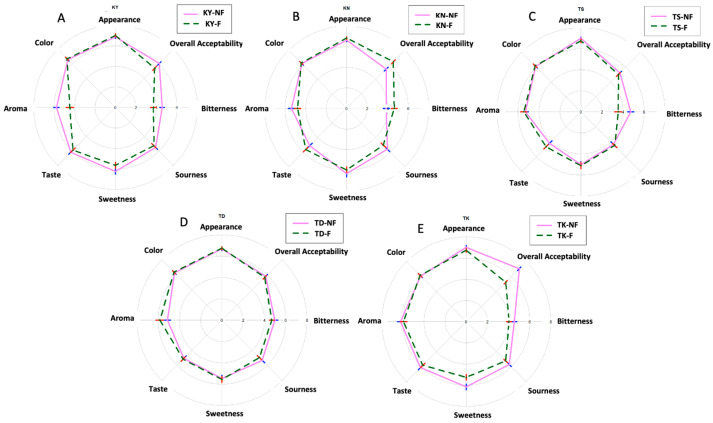
Acceptability scores of non-fermented and fermented pomelo juices from different cultivars (**A**–**E**). Data are presented as mean scores (*n* = 50). KY: Kao Yai (**A**); KN: Kao Numpueng (**B**); TS: Tubtim Siam(**C**); TD: Tong Dee (**D**); TK: Ta Koi (**E**); NF: Non-fermented; F: Fermented.

The organoleptic test of pomelo juice before and after fermentation showed significant differences in acceptance scores (Figure 2). Although appearance and color remained unchanged during fermentation, the aroma scores decreased in some cultivars despite an improvement in the aromatic compound profile. This indicates that changes in the intensity of some desirable aromatic compounds may not have been noticeable to consumers. The organoleptic assessment of the fermented samples indicated slightly lower scores for sweetness and sourness acceptance compared to the non-fermented samples. This could be attributed to the decrease in total sugar content and the increase in acidity that occurs during the fermentation process. Interestingly, the fermented juice of KN showed more improvement in terms of bitterness and overall acceptability compared to the non-fermented juice. On the other hand, no significant change in bitterness and overall acceptability was observed for TD before and after fermentation. However, the overall acceptability of both the non-fermented and fermented juice of TS did not show a significant difference. In contrast, a decrease in the acceptability scores was observed for TK and KY following fermentation.

During the fermentation of pomelo juice, there was an increase in flavonoids, particularly naringin, which contributed to an elevation in bitterness, resulting in lower acceptance scores. Similar outcomes have been reported in previous studies on fermented mango and orange juices, where fermentation by lactobacilli bacteria resulted in decreased overall liking due to an increase in sourness, bitterness, and off-flavors in the juices [9,38]. Among the non-fermented samples, KY was the most preferred juice, while KN was preferred the most among the fermented samples. These findings highlight the need to address the bitterness and optimize sweetness and sourness to enhance the consumer acceptability of fermented pomelo juice.

### 3.7. Principal Component Analysis

**Figure 3:**
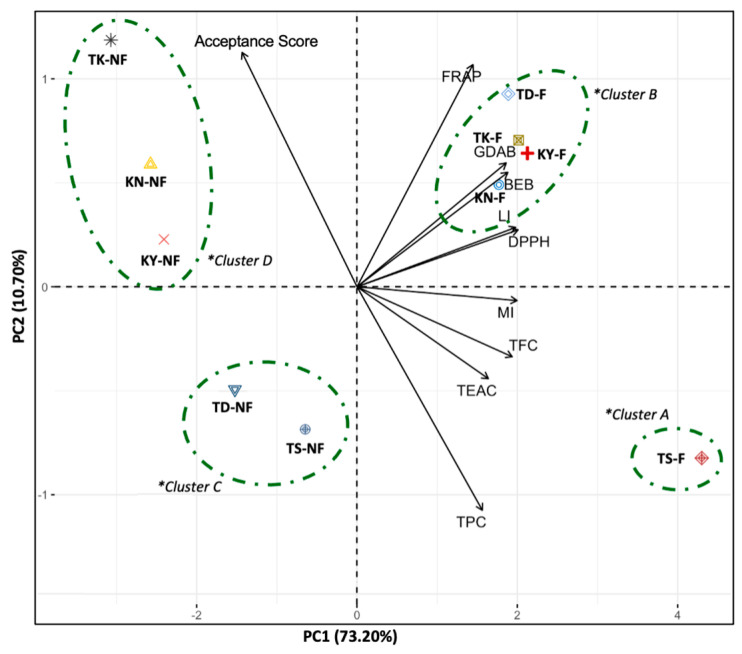
A biplot of the principal component analysis based on the phytochemical content, functional properties, and overall organoleptic acceptance scores of fermented and non-fermented pomelo juice cultivars. The data represent the average of three independent measurements. *Clusters were determined by Agglomerative Hierarchical Clustering. TS: Tubtim Siam; TD: Tong Dee; TK: Ta Koi; KN: Kao Numpueng; KY: Kao Yai; NF: Non-fermented; F: Fermented. TPC: Total Phenolic Content; TFC: Total Flavonoid Content; FRAP: ferric-reducing antioxidant power; DPPH: DPPH radical scavenging activity; TEAC: Trolox equivalent antioxidant capacity; MI: Cholesterol Micellization Inhibition; LI: Lipase activity inhibition; BEB: Bile Extract Binding; GDAB: Glycodeoxycholic acid Binding.

The principal component analysis (PCA) depicted in Figure 3 demonstrated that PC1 and PC2 explained 73.2% and 10.7% of the variance, respectively. PC1 was strongly influenced by TFC, antioxidant properties, inhibition of cholesterol micellization, pancreatic lipase inhibition, and bile acid binding capacity, while TPC and acceptability scores were major factors influencing PC2. The samples were separated by PC1 due to the increased TFC, antioxidant capacity, and functional properties of the pomelo juices after fermentation. Additionally, there was a positive relationship between the phytochemical content and functional properties of pomelo juice samples, while there was an inverse relationship between functional properties and acceptability scores. Furthermore, the agglomerative hierarchical clustering of the pomelo juice samples revealed that the fermented Tubtim Siam juice (TSF) had the highest properties and was placed in the first cluster (A). The other fermented samples (TKF, TDF, KNF, and KYF) had lower properties and were placed in the second cluster (B). The non-fermented samples were grouped into two clusters: one composed of Tubtim Siam and Tong Dee (TS and TD) with higher properties than the other cluster, composed of Ta Koi, Kao Numpueng, and Kao Yai. These results suggest that Tubtim Siam exhibited the highest properties related to health benefits and functionality among all cultivars tested in both fermented and non-fermented samples.

## 4. Conclusions

The current study provides insights into the use of different pomelo varieties for producing a probiotic beverage using *L. paracasei*. Fermentation was found to increase bioactive components and antioxidant capacity, leading to potential health benefits such as the reduction of lipid digestion which may result in a reduction of obesity and risks of cardiovascular diseases. Tubtim Siam was identified as the most suitable cultivar for producing fermented pomelo juice with desirable characteristics such as high *L. paracasei* survival, antioxidant properties, bile acid binding, lipase activity inhibition, and disruption of cholesterol micellization. This study also highlights the importance of considering cultivar variations when exploring the potential of pomelo in novel food applications. Further studies should explore probiotic viability, phytochemical bioaccessibility during storage and digestion, and the effect on the gut microbiota to establish the functionality and health benefits of fermented pomelo beverages.

## Figures and Tables

**Table 1:** pH and organic acids of pomelo juices before and after 24 h fermentation.

Samples		pH	Organic Acids (mg/mL)					
			Tartaric Acid	Ascorbic Acid	Citric Acid	Lactic Acid	Acetic Acid	Total Organic Acids
KN	NF	4.56 ± 0.12 ^abA^	0.03 ± 0.00 ^deB^	0.10 ± 0.01 ^bcA^	1.50 ± 0.03 ^cdA^	0.0 ^cB^	0.0 ^eB^	1.40 ± 0.03 ^cB^
	F	4.49 ± 0.18 ^aA^	0.06 ± 0.02 ^bcA^	0.10 ± 0.01 ^bcA^	1.27 ± 0.21 ^bcA^	2.77 ± 0.26 ^bA^	0.43 ± 0.04 ^aA^	4.90 ± 0.85 ^aA^
KY	NF	4.53 ± 0.16 ^abA^	0.03 ± 0.00 ^deB^	0.08 ± 0.00 ^cdA^	1.22 ± 0.19 ^cdA^	0.0 ^cB^	0.0 ^eB^	1.69 ± 0.19 ^bB^
	F	4.37 ± 0.01 ^abA^	0.08 ± 0.01 ^abA^	0.10 ± 0.01 ^bcA^	1.15 ± 0.16 ^cdA^	3.65 ± 0.33 ^aA^	0.19 ± 0.04 ^cA^	5.12 ± 0.29 ^aA^
TK	NF	4.57 ± 0.03 ^bA^	0.08 ± 0.01 ^abA^	0.06 ± 0.01 ^dB^	2.13 ± 0.16 ^aA^	0.0 ^cB^	0.0 ^eB^	2.63 ± 0.24 ^bB^
	F	4.44 ± 0.02 ^aA^	0.02 ± 0.01 ^dfB^	0.08 ± 0.01 ^cdA^	1.28 ± 0.06 ^cdB^	2.89 ± 0.21 ^bA^	0.29 ± 0.06 ^bA^	4.31 ± 0.69 ^aA^
TD	NF	4.46 ± 0.11 ^bA^	0.09 ± 0.01 ^aA^	0.13 ± 0.02 ^abA^	1.15 ± 0.15 ^cdA^	0.0 ^cB^	0.0 ^eB^	1.50 ± 0.17 ^bcB^
	F	4.27 ± 0.17 ^aA^	0.09 ± 0.01 ^aA^	0.10 ± 0.01 ^bcA^	1.01 ± 0.04 ^dA^	2.85 ± 0.21 ^bA^	0.17 ± 0.05 ^cA^	5.39 ± 0.31 ^aA^
TS	NF	4.49 ± 0.05 ^aA^	0.05 ± 0.01 ^cdA^	0.17 ± 0.00 ^aA^	1.81 ± 0.04 ^abA^	0.0 ^cB^	0.0 ^eB^	2.12 ± 0.01 ^bB^
	F	4.26 ± 0.15 ^aA^	0.06 ± 0.01 ^bcA^	0.14 ± 0.01 ^aA^	0.98 ± 0.03 ^dB^	3.13 ± 0.26 ^abA^	0.09 ± 0.02 ^deA^	4.40 ± 0.29 ^aA^

Data are expressed as Mean ± SEM, *n* = 3. Means with different uppercase letters (fermentation effects) at the same cultivar are significantly different (*p* < 0.05). Means with different lowercase letters (treatment effects) are significantly different (*p* < 0.05). Abbreviations: KN: Kao Numpueng; KY: Kao Yai; TK: Ta Koi; TD: Tong Dee; TS: Tubtim Siam; NF: Non-fermented juice; F: Fermented juice.

**Table 2:** Total sugar, reducing sugar, color values, and viable lactic acid bacteria count of pomelo juices before and after 24 h fermentation.

Samples		Total Sugar (mg/mL)	Reducing Sugar (mg/mL)	Color Values				Lactic Acid Bacteria Count (log cfu/mL)
				L*	a*	b*	∆E	
KN	NF	109.04 ± 1.56 ^abA^	13.54 ± 0.02 ^abA^	27.79 ± 0.47 ^bB^	−3.15 ± 0.15 ^cA^	−2.67 ± 0.29 ^cdA^	4.53 ± 0.41	7.09 ± 0.50 ^aB^
	F	98.58 ± 3.39 ^bB^	10.83 ± 0.79 ^bB^	32.29 ± 0.76 ^bA^	−2.78 ± 0.06 ^cA^	−3.09 ± 0.02 ^dA^		8.86 ± 0.48 ^abA^
KY	NF	124.5 ± 3.50 ^aA^	14.33 ± 0.09 ^aA^	29.33 ± 0.30 ^abB^	1.67 ± 0.13 ^bB^	−3.71 ± 0.24 ^dA^	4.53 ± 0.01	7.08 ± 0.01 ^aB^
	F	120.40 ± 9.9 ^aB^	8.50 ± 0.43 ^cB^	33.77 ± 0.66 ^abA^	2.45 ± 0.06 ^bA^	−4.15 ± 0.16 ^eA^		9.12 ± 0.51 ^aA^
TK	NF	96.88 ± 6.88 ^bA^	12.46 ± 0.05 ^bA^	27.40 ± 0.79 ^bB^	−3.41 ± 0.13 ^cA^	−1.67 ± 0.19 ^bcA^	4.84 ± 0.23	7.06 ± 0.05 ^aB^
	F	92.45 ± 4.16 ^bB^	9.76 ± 0.09 ^bcB^	32.21 ± 0.48 ^bA^	−3.04 ± 0.07 ^cA^	−2.02 ± 0.33 ^cA^		8.80 ± 0.52 ^bA^
TD	NF	101.87 ± 0.39 ^abA^	12.27 ± 0.13 ^bA^	28.52 ± 1.10 ^abB^	1.52 ± 0.10 ^bB^	−1.65 ± 0.52 ^bA^	5.26 ± 0.16	7.08 ± 0.15 ^aB^
	F	92.55 ± 3.33 ^abB^	11.02 ± 0.07 ^bcB^	33.73 ± 0.29 ^abA^	2.25 ± 0.25 ^bA^	−1.82 ± 0.57 ^bA^		9.01 ± 0.17 ^abA^
TS	NF	126.19 ± 6.29 ^aA^	14.41 ± 0.09 ^aA^	32.53 ± 1.35 ^aB^	4.25 ± 0.10 ^aB^	1.54 ± 0.22 ^aA^	4.31 ± 0.13	7.09 ± 0.02 ^aB^
	F	112.8 ± 4.71 ^abB^	8.44 ± 0.34 ^cB^	36.71 ± 0.84 ^aA^	5.29 ± 0.07 ^aA^	1.67 ± 0.04 ^aA^		9.28 ± 0.30 ^aA^

Data are expressed as Mean ± SEM, *n* = 3. Means with different uppercase letters (fermentation effects) at the same cultivar are significantly different (*p* < 0.05). Means with different lowercase letters (treatment effects) are significantly different (*p* < 0.05). Abbreviations: KN: Kao Numpueng; KY: Kao Yai; TK: Ta Koi; TD: Tong Dee; TS: Tubtim Siam; NF: Non-fermented juice; F: Fermented juice.

**Table 3:** Phytochemical and antioxidant properties of pomelo juices before and after 24 h fermentation.

Samples		TPC (μg GAE/mL)	TFC (μg QE/mL)	FRAP (mmol FeSO_4_/mL)	TEAC (mg Trolox Eq/mL)	DPPH (mg AAE/mL)
KN	NF	910.76 ± 2.06 ^bcB^	25.21 ± 0.09 ^gB^	8.71 ± 0.18 ^cB^	1.70 ± 0.08 ^bB^	0.29 ± 0.06 ^cB^
	F	963.89 ± 3.16 ^bcA^	54.00 ± 0.26 ^dA^	10.56 ± 0.08 ^bcA^	1.94 ± 0.03 ^abA^	0.82 ± 0.02 ^abA^
KY	NF	913.43 ± 1.07 ^bcB^	31.87 ± 0.27 ^fB^	9.81 ± 0.28 ^bcB^	1.56 ± 0.04 ^bcB^	0.26 ± 0.02 ^bB^
	F	1053 ± 6.09 ^aA^	56.92 ± 0.46 ^cA^	10.96 ± 0.29 ^abA^	1.76 ± 0.01 ^bA^	0.64 ± 0.12 ^bA^
TK	NF	893.16 ± 3.07 ^cB^	31.43 ± 0.26 ^fB^	8.46 ± 0.11 ^cB^	1.42 ± 0.06 ^cB^	0.23 ± 0.01 ^cB^
	F	962.78 ± 2.09 ^bcA^	65.22 ± 0.17 ^bA^	9.45 ± 0.19 ^bcA^	1.73 ± 0.02 ^bA^	0.93 ± 0.05 ^aA^
TD	NF	957.45 ± 2.01 ^bcB^	34.50 ± 0.18 ^eB^	8.82 ± 0.24 ^cB^	1.75 ± 0.02 ^bB^	0.27 ± 0.02 ^cB^
	F	1037.2 ± 3.02 ^abA^	54.45 ± 0.74 ^dA^	11.57 ± 0.31 ^abA^	1.85 ± 0.02 ^bA^	0.92 ± 0.03 ^aA^
TS	NF	1063.14 ± 1.06 ^abB^	81.71 ± 0.16 ^bB^	11.85 ± 0.42 ^abB^	2.21 ± 0.03 ^aB^	0.34 ± 0.03 ^cB^
	F	1288.67 ± 3.05 ^aA^	118.99 ± 0.16 ^aA^	13.06 ± 0.28 ^aA^	2.56 ± 0.08 ^aA^	0.98 ± 0.06 ^aA^

Data are expressed as Mean ± SEM, *n* = 3. Means with different uppercase letters (fermentation effects) at the same cultivar are significantly different (*p* < 0.05). Means with different lowercase letters (treatment effects) are significantly different (*p* < 0.05). Abbreviations: KN: Kao Numpueng; KY: Kao Yai; TK: Ta Koi; TD: Tong Dee; TS: Tubtim Siam; NF: Non-fermented juice; F: Fermented juice: TPC: Total Phenolic Content; TFC: Total Flavonoid Content; FRAP: ferric-reducing antioxidant power; DPPH: DPPH radical scavenging activity; TEAC: Trolox equivalent antioxidant capacity (TEAC).

**Table 4:** Flavonoid constituents of fermented and non-fermented pomelo juices.

Samples		Naringin (μg/g)	Hesperidin (μg/g)	Neohesperidin (μg/g)	Naringenin (μg/g)	Hesperetin (μg/g)
KN	NF	442.80 ± 7.59 ^cB^	77.57 ± 2.34 ^bA^	41.21 ± 11.66 ^bA^	1.86 ± 0.23 ^bcB^	0.51 ± 0.03 ^deB^
	F	1271.59 ± 15.34 ^abA^	51.81 ± 9.74 ^bA^	67.51 ± 13.89 ^bA^	3.19 ± 0.62 ^bA^	2.25 ± 0.08 ^abcA^
KY	NF	208.99 ± 9.48 ^cB^	45.78 ± 5.81 ^bB^	56.83 ± 6.54 ^bA^	0.53 ± 0.0.06 ^cA^	0.33 ± 0.03 ^eB^
	F	472.19 ± 5.27 ^cA^	71.33 ± 8.46 ^bA^	52.92 ± 5.45 ^bA^	0.81 ± 0.07 ^cA^	0.86 ± 0.04 ^bcdeA^
TK	NF	1114.91 ± 32.12 ^bcA^	217.85 ± 15.13 ^abA^	99.21 ± 12.41 ^abB^	1.00 ± 0.03 ^cA^	0.38 ± 0.01 ^eA^
	F	1291.22 ± 19.42 ^abcA^	237.79 ± 18.06 ^abA^	144.91 ± 23.08 ^aA^	1.76 ± 0.08 ^bcA^	0.58 ± 0.05 ^cdeA^
TD	NF	710.77 ± 7.38 ^cA^	333.98 ± 7.74 ^aB^	73.69 ± 4.68 ^bB^	1.18 ± 0.05 ^cA^	1.66 ± 0.05 ^bcdeB^
	F	717.54 ± 5.02 ^cA^	460.09 ± 20.30 ^aA^	105.68 ± 12.44 ^abA^	1.95 ± 0.04 ^cA^	2.31 ± 0.08 ^abA^
TS	NF	2271.38 ± 43.04 ^abA^	403.75 ± 8.65 ^aA^	113.30 ± 9.13 ^abA^	3.48 ± 0.06 ^bB^	2.12 ± 0.24 ^abcdA^
	F	2517.00 ± 30.51 ^aA^	263.97 ± 4.14 ^abA^	61.67 ± 5.84 ^bB^	6.66 ± 0.78 ^aA^	3.33 ± 0.15 ^aA^

Data are expressed as Mean ± SEM, *n* = 3. Means with different uppercase letters (fermentation effects) at the same cultivar are significantly different (*p* < 0.05). Means with different lowercase letters (treatment effects) are significantly different (*p* < 0.05). Abbreviations: KN: Kao Numpueng; KY: Kao Yai; TK: Ta Koi; TD: Tong Dee; TS: Tubtim Siam; NF: Non-fermented juice; F: Fermented juice.

**Table 5:** Volatile flavor compounds identified in pomelo juices.

Compound	Tentative Identification	Odor Description	Peak Area (×10^2^)									
			KN		KY		TD		TK		TS	
			NF	F	NF	F	NF	F	NF	F	NF	F
	***Alcohols***											
1	1-Hexanol	Green, Fruity	N.D.	N.D.	N.D.	N.D.	N.D.	N.D.	2.84 ± 0.15 ^A^	1.69 ± 0.09 ^A^	4.08 ± 1.36 ^A^	1.23 ± 0.13 ^B^
2	1-Octen-3-ol	Earthy, Herbaceous	1.68 ± 0.35 ^A^	1.05 ± 0.06 ^A^	N.D.	N.D.	1.17 ± 0.03 ^A^	1.72 ± 0.03 ^A^	N.D.	N.D.	N.D.	N.D.
	***Aldehydes***											
3	(*Z*)-3-Hexenal	Green, Leafy	N.D.	N.D.	3.09 ± 0.16 ^A^	2.83 ± 0.15 ^A^	N.D.	N.D.	7.73 ± 1.190	N.D.	15.67 ± 1.99	N.D.
4	2,4-Decadienal, (*E*,*E*)-	Citrus, Green	5.66 ± 0.81 ^A^	3.45 ± 0.5 ^B^	3.85 ± 0.93 ^A^	1.46 ± 0.81 ^B^	1.27 ± 0.97	N.D.	4.71 ± 0.52 ^A^	3.54 ± 0.44 ^A^	7.12 ± 1.92	N.D.
	***Esters***											
5	Ethyl acetate	Acidic, Sweet	5.91 ± 0.59 ^B^	17.13 ±1.26 ^A^	5.74 ± 0.08 ^A^	5.69 ± 0.03 ^A^	9.07 ± 1.71 ^B^	30.66 ± 1.31 ^A^	7.86 ± 0.04 ^A^	7.73 ± 0.10 ^A^	11.10 ± 0.10 ^A^	11.14 ± 0.06 ^A^
	***Hydrocarbons***											
6	Myrcene	Lemon, Musty	4.03 ± 0.57 ^A^	1.22 ± 0.26 ^B^	N.D.	N.D.	1.31 ± 0.43	N.D.	24.58 ± 2.70 ^A^	18.38 ± 1.80 ^B^	N.D.	N.D.
7	(+)-alpha-Phellandrene	Citrus, Green	8.94 ± 1.88 ^A^	6.48 ± 1.25 ^B^	N.D.	N.D.	N.D.	N.D.	N.D.	N.D.	N.D.	N.D.
8	L-Limonene	Citrus, Terpenic	37.69 ±1.75 ^A^	27.91 ± 1.29 ^B^	13.78 ± 0.11 ^A^	4.12 ± 0.15 ^B^	14.60 ± 0.10 ^A^	2.56 ± 0.14 ^B^	11.74 ± 0.13 ^A^	1.19 ± 0.07 ^B^	12.94 ± 1.01 ^A^	9.07 ± 0.49 ^B^
	***Ketones***											
9	Butan-2-one	Cheese, Ether-like	N.D.	62.75 ± 2.37	N.D.	121.52 ± 3.46	N.D.	92.70 ± 1.65	N.D.	66.80 ± 7.60	N.D.	165.92 ± 9.78
10	Acetoin	Creamy, Buttery	N.D.	26.98 ± 1.88	N.D.	2.82 ± 0.50	N.D.	1.98 ± 0.14	N.D.	2.37 ± 0.64	N.D.	3.38 ± 0.64
11	3,5-Octadien-2-one	Fruity, Mushroom	N.D.	28.02 ± 2.97	N.D.	23.09 ± 2.47	N.D.	5.04 ± 0.49	N.D.	21.02 ± 2.72	N.D.	11.47 ± 1.81
	***Others***											
12	Indole	Floral, Sweet	N.D.	N.D.	2.65 ± 0.38 ^A^	3.87 ± 0.64 ^A^	N.D.	N.D.	1.60 ± 0.32	N.D.	1.39 ± 0.34 ^A^	1.34 ± 0.23 ^A^
13	bis(2-furylmethyl) sulfide	Green, Earthy	13.57 ± 0.36 ^A^	8.41 ± 0.14 ^B^	14.58 ± 0.44 ^A^	9.61 ± 1.53 ^B^	12.87 ± 3.64 ^A^	5.14 ± 0.94 ^B^	15.88 ± 2.52 ^A^	8.89 ± 0.27 ^B^	19.43 ± 3.83 ^A^	7.45 ± 1.79 ^B^

The peak areas are obtained from the MXT-1701 column. Data are expressed as Mean ± SEM, *n* = 3. Means with different uppercase letters (fermentation effects) at the same cultivar are significantly different (*p* < 0.05). Abbreviations: KN: Kao Numpueng; KY: Kao Yai; TK: Ta Koi; TD: Tong Dee; TS: Tubtim Siam; NF: Non-fermented juice; F: Fermented juice.

## Data Availability

Data are contained within the article or Supplementary Materials.

## Supplementary Materials

The following supporting information can be downloaded at: https://www.mdpi.com/article/10.3390/foods12234278/s1, Table S1, Retention time (RT) and Kovats retention indices (KI) for alkane standards C6-C16. Table S2, Retention time (RT) and Kovats retention indices (KI) for identified volatile compounds.
